# Evaluating Technology-Based Self-Monitoring of Performance with Differential Reinforcement for Students with Disabilities

**DOI:** 10.3390/bs13060508

**Published:** 2023-06-18

**Authors:** Madeline R. Risse, Kwang-Sun Cho Blair, Danielle A. Russo

**Affiliations:** Applied Behavior Analysis Program, Department of Child and Family Studies, University of South Florida, Tampa, FL 33612, USA; kwangsun@usf.edu (K.-S.C.B.); russod@usf.edu (D.A.R.)

**Keywords:** technology, self-monitoring, school-based intervention, behavior, inclusion

## Abstract

This study evaluated the use of a technology-based self-monitoring of performance (SMP) with differential reinforcement to increase task completion and reduce off-task behavior for three 5th-grade students with disabilities. A concurrent multiple baseline design across participants was used to examine the impact of the intervention on the targeted behaviors when implemented by a general education teacher and its maintenance effects with a delay of reinforcement. The implementation involved training students to use a mobile app for SMP and providing differential reinforcement contingent on task completion and accuracy of self-monitoring during academic periods. The secondary measure of off-task behavior was included to evaluate the relationship between task completion and engagement. The results demonstrated that the technology-based SMP with differential reinforcement increased task completion and reduced off-task behavior for all students. Furthermore, the gradual fading of the reinforcement, with a 45 min delay, was successful for all students. The efficiency and immediacy of the intervention suggest that technology-based SMP with differential reinforcement holds promise as a practical, efficient, and effective school-based intervention.

## 1. Introduction

Self-regulation skills encompass the ability to monitor and modify one’s own behavior, emotions, or thoughts in response to the changing demands of an environment, context, or situation [1,2] Researchers have shown that self-regulation skills are a critical predictor of academic achievement; however, certain students who struggle with inattention, impulsivity, and inhibition control may encounter difficulties in developing these skills [2,3,4]. Particularly, students with disabilities often have difficulty self-regulating their social and academic behaviors, which has been consistently associated with diminished academic productivity, compromised social competence, and heightened teacher frustration [3,4,5,6]. Unless specific interventions are implemented, these students are unlikely to meet the social and academic expectations within a general education setting [7,8]. 

Students with disabilities who have limited self-regulating abilities may encounter difficulties in attending to tasks, engaging in appropriate social interactions, and maintaining positive behaviors in the classroom. These challenges can pose difficulties for teachers to effectively manage the classroom while maintaining instructional control [9,10]. Thus, when problem behavior occurs at a level that exceeds what teachers can proficiently manage in the classroom, inclusion for students with disabilities becomes increasingly difficult to sustain [11]. As students with disabilities increasingly receive services in the general education setting, it is paramount that efficient, feasible, and contextually fit evidence-based practices (EBPs) are available for use by general education teachers [7,12,13]. Unfortunately, general education teachers are less likely to accurately implement EBPs to address the problem behavior in their classrooms, and often report feeling unprepared to address the needs of students with disabilities [9,10,11,14]. To address the research-to-practice gap, researchers have emphasized the critical nature of teacher training to increase teachers’ ability to proficiently implement EBPs, especially those which ameliorate social and academic deficits concurrently [9,13]. 

### 1.1. Self-Monitoring

Self-monitoring (SM) is a key component of self-management strategies used to improve students’ self-regulation abilities whereby an individual observes and records their own behavior to assess whether a target behavior has occurred [15,16]. Theoretically, when using SM, students must discriminate the occurrence of a target behavior and then self-record some dimension of the target behavior, resulting in improvements to their self-regulation skills [16,17]. As an EBP, SM has been used to support a variety of students displaying problem behavior across grade levels, requiring minimal investment of teacher time and resources [18]. Self-monitoring has been used to increase appropriate behavior (e.g., on-task behavior, task completion, academic performance, instructional engagement) and decrease problem behavior that impacts student learning [8,19,20,21,22,23]. 

Using SM in the school setting offers multiple benefits, such as producing an immediate and impactful change in behavior, being cost-effective, relatively unobtrusive, time-efficient, and easily individualized to fit the individual students’ needs, teachers’ instructional styles, logistics of recording, and availability of resources [5,12]. In culmination, these benefits minimize the loss of instructional time during implementation which can free up time for other classroom responsibilities and result in a reduction of teacher stress [8,21]. Social validity assessments have indicated that teachers found SM interventions to be feasible, appropriate, and effective within the classroom context [24,25]. The SM intervention is a proactive strategy that facilitates the inclusion of students with disabilities in general education settings because the intervention is implemented in the classroom where the target behavior typically occurs, thus eliminating the need to remove the students from the learning environment [5,21]. As such, teachers may implement practical strategies such as SM to improve student behavior and make it less likely that they will be suspended, fail courses, or even drop out [5].

### 1.2. Self-Monitoring of Performance 

Researchers have commonly examined the use of self-monitoring of attention (SMA) and self-monitoring of performance (SMP) to help students self-manage their behaviors [3,6,16]. The focus of SMA is on increasing students’ awareness of their attentive behaviors (e.g., on-task or off-task) by instructing them to monitor and record some form of their attending behavior when cued at predetermined intervals [12,16]. Conversely, the focus of SMP is increasing academic performance and task-related responding by instructing students to monitor some aspect of their academic performance (e.g., completion or accuracy) during or following a task [12,13,16]. Proponents of SMP emphasize that although academic productivity will affect attentional behavior, attentional behavior may not necessarily affect academic productivity [6,13]. 

The SMP intervention can be individualized to meet a variety of student and teacher needs with little investment of time or resources while offering a variety of benefits to students, including greater independence in the classroom, increased academic performance, and improved self-management skills [13,21,26]. The literature reports that SMP is effective in not only increasing task completion and on-task behavior, but also improving academic performance exhibited by a variety of student populations when teachers implement it with high fidelity [13,27]. For example, Barry and Messer [27] evaluated using an SMP intervention in a 6th-grade general education classroom with five students with attention deficit hyperactivity disorder (ADHD). The SMP included monitoring academic performance, on-task behavior, and disruptive behavior. The SMP resulted in increased task completion, academic performance, and on-task behavior, and decreased disruptive behavior for all students. Rafferty and Raimondi [13] examined using both SMA and SMP with three middle school students with EBD served in an inclusive general education classroom. The findings revealed that SMP resulted in higher levels of social and academic behaviors compared to SMA. Specifically, students using SMP exhibited on-task behavior comparable to that of typical peers, demonstrated greater academic productivity and accuracy, and more significant reductions in off-task behavior. Notably, regardless of the order of implementation, each student consistently selected SMP during the choice condition, indicating a preference for the SMP over the SMA. This preference suggests that teaching students to self-monitor their academic performance may yield more desirable outcomes than teaching them to self-monitor their attentional behavior. However, due to limited existing evidence on the effectiveness of SMP, further research is needed to examine the potential benefits of using SMP in improving student behavior and enhancing academic performance.

### 1.3. Self-Monitoring with Differential Reinforcement

The existing body of research on school-based SM interventions has well-documented varying effects of supplementary components or materials incorporated into SM intervention on student behavior [5,18]. Typically, the most effective SM interventions are multi-component in nature and include reinforcement and/or feedback linked to student performance [5]. In comparison to noncontingent reinforcement, contingent reinforcement directed at desired behaviors, such as accurate recording, meeting a goal, or completing a task, has yielded greater behavioral outcomes [18]. For example, Bruhn and Watt [28] evaluated an SM intervention package involving two middle school students, who self-rated their adherence to multiple classroom expectations. At the end of each session, reinforcement was provided contingent upon the students obtaining 80% of the maximum possible points during the work period. The SM intervention resulted in increased academic engagement and decreased disruptive behavior for both participants. It has been suggested that extinction is not a required component of differential reinforcement, and in situations where extinction may not be possible, manipulating the quality, duration, immediacy, and magnitude of reinforcement for an alternative or desired behavior may offer distinct advantages [29].

In a review conducted by Briesch et al. [30], most of the studies examined required students to self-observe and record their own behavior without receiving external feedback or reinforcement; however, nearly all studies employed frequent recording of behavior, prompting students to self-assess at least every 2 min during a task. Considering that students may find the frequent prompted monitoring during a task to be more intrusive and less motivating than monitoring after the task [31], it remains unclear whether frequent, prompted SM leads to sustainable changes in student behavior. Furthermore, Mize et al. [32] recommended that future researchers investigate SM applications incorporating effective instructional components, such as reinforcement and error correction delivered by a teacher. Further research is necessary to evaluate whether SMP interventions that involve reinforcement without prompts and include teacher-delivered instructional components (i.e., reinforcement and error correction as needed) will yield similar behavioral improvements for students with disabilities, and whether these behavior changes will maintain over time. 

### 1.4. Self-Monitoring with Technology 

Until recently, most SM procedures and recording devices were characterized by their simplicity and low-tech nature, relying on materials such as paper, pencil, timers, and buzzers to record student behavior [17,32]. However, utilizing such materials can produce social stigmatization, lead to embarrassment for students, and pose a greater investment of teacher time and materials to prepare [33]. Studies have reported that paper and pencil strategies are less acceptable to students, and lack social validity [34]. To address these limitations, the adoption of mobile-technology-based SM interventions has emerged as a promising alternative. The use of the technology provides greater portability and convenience, and recent research has demonstrated its effects on improving the on-task behaviors of students with disabilities [32]. Several studies conducted in schools have shown positive outcomes when utilizing mobile technology for SM [24,33]. For example, Wills and Mason [23] utilized a handheld tablet to teach two high school students with disabilities to self-monitor attention in a general education setting. The SM intervention increased on-task behavior for both students. Bedesem [24] evaluated an SM intervention procedure with two middle school students utilizing cell phones with the CellFM app to record SM data. The intervention had positive impacts on both students’ on-task behavior, and socially valid data indicated high acceptability of the intervention by students and teachers alike. These results are in alignment with previous research suggesting that handheld technology may be more appealing and socially acceptable than typical monitoring methods, piquing the interest and improving the buy-in of students participating [26,32]. 

With rapidly developing technologies available for use by teachers in school-based settings, Mize et al. [32] urged future researchers to examine up-to-date mobile technology (e.g., iPhone, iPod) to deliver SM interventions to improve student behaviors. The continuous progress of 21st-century technology and the growing demand for effective virtual learning strategies, necessitate further investigation into the use of technology-based SM intervention applications in schools [15]. More specifically, given the positive effects of technology-based SM and findings that indicate screen size does not impede the effectiveness of the intervention, researchers should investigate whether technology-based SMP, utilizing a mobile app as a recording medium, yields similar or greater results than traditional methods [32]. Moreover, in school settings, implementing extinction procedures with fidelity may not be feasible for educators. Consequently, interventions designed for use in classroom settings may focus on providing increased reinforcement contingent upon the occurrence of an alternative response, aiming to shift response allocation across two or more alternatives when problem behavior continues to contact unprogrammed reinforcement.

### 1.5. Maintenance Effects of Self-Monitoring

Researchers have noted that SM with reinforcement is likely to reduce the time required to improve student behavior [21]. However, the literature provides limited information on the sustainability of behavior change when SM components are faded or guidance for practitioners on how to plan for maintenance [30]. Bruhn and colleagues [18] reported that only eight studies of the 41 studies they reviewed included generalization probes and only nine included maintenance programming. This is detrimental when teachers are responsible for implementation without other professionals guiding them to systematically fade intervention components. Future researchers should evaluate strategies to train teachers to systematically fade intervention components in school-based settings while formally evaluating student behavior. This can ensure that intervention effects maintain beyond implementation while capitalizing on the utility of technology to support intervention delivery and data-based decision-making [26]. Future research on SMP should involve training teachers to make data-driven intervention decisions that promote sustained behavior change. 

### 1.6. Current Study

The SM intervention has been effective when utilized to support students with disabilities in a variety of settings, including inclusive classrooms (e.g., [6,23,24,27]), self-contained classrooms or resource rooms (e.g., [13,28,33]), and alternative schools (e.g., [22,29]). Given the positive effects of using up-to-date mobile technology to improve the on-task behavior of students with disabilities, more research is needed to identify practical and feasible technology-based SM applications that are palatable to both students and teachers [32]. The current literature indicates that technology-based SMP may offer a modern way to deliver behavioral intervention to support students with disabilities in the general education setting while capitalizing on the efficiency of technology and keeping students in the classroom [5]. Furthermore, experimental research examining the effects of technology-based SM is crucial [15]. Specifically, few studies have attempted to examine SMP interventions that utilize technology as a recording medium, differential reinforcement, independent use of a checklist in the absence of prompts or devices, and evaluation of its maintenance effects.

To fill the gaps in the literature, the current study aimed to evaluate the use of a technology-based SMP with contingent reinforcement for task completion and accuracy of SM for students with disabilities served in the inclusive 5th-grade general education setting. The study addressed the following research questions: (a) to what extent will the implementation of a technology-based SMP with differential reinforcement increase task completion and reduce off-task behavior in students with disabilities served in inclusive general education classrooms, (b) to what extent will the effects of SMP intervention be maintained after the fading procedure is complete, and (c) to what extent will improvements in the target behaviors be maintained without intervention at 4-week follow-up?

## 2. Materials and Methods

### 2.1. Participants and Setting

Three students in fifth grade (10 or 11 years old) and one corresponding general education teacher participated in the study. Inclusionary criteria for students included: (a) special education eligibility for an identified disability, (b) enrollment in the general education setting for the majority (51% or more) of the school day, (c) history of off-task behavior throughout regular classroom activities, (d) teacher identification of chronic difficulties maintaining adequate levels of task completion (e.g., frequent incomplete or missing assignments), and (e) willingness of the teacher to participate in training and implement the SMP intervention. Exclusionary criteria for students included: (a) chronic absenteeism defined by accumulating four or more absences within the past calendar month, (b) problem behavior too severe to manage in the classroom (e.g., requiring removal from the learning environment for safety), and (c) current or previous training or use of an SMP procedure in the classroom.

#### 2.1.1. Students

Dominic was an 11-year-old boy, white, in 5th grade diagnosed with attention deficit hyperactive disorder (ADHD), who was eligible for special education services (SES) under the categories of other health impairment (OHI) and speech and language impairment (LI). Dominic’s teacher reported that he typically completed approximately 10% of classroom tasks and engaged in off-task behavior approximately 80% of the time each class period prior to the study. Examples of his typical off-task behaviors included fidgeting with materials in his desk, using pencils to “drum” on his desk, and gazing around the room or out of the window. Dominic’s teacher reported concerns about his academic progress in reading and writing due to the occurrence of his off-task behavior and failure to complete most classroom assignments. 

Selena was a 10-year-old girl, Black, in 5th grade, who was diagnosed with ADHD and was eligible for SES under the categories of OHI and specific learning disability (SLD). Selena was described by her teacher to be easily distracted by social interactions in the classroom. Selena’s teacher reported that she completed approximately 60% of classroom tasks and engaged in off-task behavior approximately 40% of the time prior to the study. Examples of typical off-task behavior for Selena included walking around the classroom without permission, engaging with personal materials interfering with tasks (backpack, pencil pouch), and talking to peers. Although Selena was slightly below the grade level academically, the teacher reported that her grades and test scores often do not reflect her true academic capability.

Robin was a 10-year-old girl, Hispanic, in 5th grade, who was eligible for SES under the categories of SLD and LI. Robin was described by her teacher to be somewhat disengaged, well below grade level, and relatively resistant to academic interventions utilized in the past. Robin’s teacher reported that she completed approximately 50% of classroom tasks and engaged in off-task behavior approximately 50% of the time prior to the study. Examples of off-task behavior for Robin included whispering with peers, gazing around the room, head down, or fidgeting with hands or nails. Her teacher reported concerns about her academic performance and lack of engagement with classroom tasks or instruction.

#### 2.1.2. Teachers

The teacher participant was a 58-year-old woman, white, with a master’s degree and 33 years of teaching experience. The teacher worked as a 5th grade English Language Arts (ELA) teacher and held certifications in elementary education (grades 1–6), varying exceptionalities (grades k-12), and social studies (grades 6–12), as well as endorsements in pre-kindergarten instruction and ESOL.

#### 2.1.3. Setting

The study was conducted in an inclusive 5th-grade general education classroom at a public elementary school in Florida, during regular instructional routines. The SMP intervention was implemented during two consecutive academic periods instructed by the same teacher in the same classroom, presenting the same instruction and tasks to a maximum of 25 students at a time. Sessions were conducted 2–3 times per week during the first 30 min of ELA and Writing. The general education teacher, who typically presented tasks during the academic period, implemented the SMP intervention. The expectation for students during the academic time was to work on assigned tasks for the entire 30 min, with the teacher being available for questions. If students finished before the 30 min concluded, they were instructed to review their work for quality and accuracy and make revisions as necessary. Example tasks presented included independent writing assignments, grammar worksheets, or reading response journals.

### 2.2. Materials

The materials for the study included three iPhones without cellular capability pre-loaded with the free List iPhone app. The authors of this study have no affiliation or conflict of interest related to the app or its creators. The List app (http://www.junglegymlabs.com/ [accessed on 15 July 2021]) was used as a recording device for students during the SMP intervention. The decision to use the free version of the List app was based on its easy accessibility, that it was free to download, and that it had a user-friendly interface. During in vivo observations, it was noted that the app offered limited navigation options thereby reducing the likelihood of students engaging in off-task behaviors while using the app, such as navigating to other screens and engaging with ads). During the intervention, student participants completed classroom tasks and checked the box next to each item on the List app as they completed the corresponding task. The app continuously updates to display the completion rate (e.g., three of four tasks completed) as it is used. The teacher also used a debriefing form, points system flowchart, and reinforcer menu when meeting with students following each intervention session, as well as a pocket guide reference of the SMP procedures provided during training. The research team utilized the SM checklist, off-task recording form, implementation fidelity checklist, and a mobile timer to collect data. Fidelity checklists were used during training to ensure the accurate implementation of training procedures.

Students completed a 10 min indirect preference assessment in the form of a reinforcer questionnaire prior to the baseline that was used to identify backup reinforcers to utilize in correspondence with the points system during the intervention. The reinforcer questionnaire consisted of five open-ended questions and a checklist of commonly preferred items and activities. Students completed the open-ended questions and circled the most preferred items and activities from the checklist. The teacher-reviewed student responses to determine preferred items to include within the reinforcer menu based on the availability of items and feasibility of delivery. The information was used to identify a variety of backup reinforcers (e.g., edibles, drawing activities, sensory toys) to use as reinforcement for student participants during the intervention. The derived reinforcer menu consisted of individualized differential reinforcement options at each leveled category that students could choose from based on the number of points earned.

### 2.3. Measurements 

#### 2.3.1. Student Behavior

This study measured two student target behaviors: task completion and off-task behavior. Task completion was the primary dependent variable and was defined as the completion of assigned academic tasks as identified in the task instructions or determined by the teacher. The research team, consisting of two graduate students, measured task completion using permanent products of student work samples completed throughout the session and a checklist of all tasks presented throughout the session to determine the percentage of task completion for each session. The percentage of tasks completed was calculated by counting the number of problems or tasks on the task list that were completed and dividing the completed number of tasks by the total number of tasks presented throughout the session and multiplying by 100. During the intervention, the research team validated the task completion measure after each session by reviewing the work samples and SM task list to determine the percentage of task completion following the teacher debriefing. The student SM task completion percentage, teacher-validated task completion percentage, and research assistant (RA) task completion percentage were recorded for each session to ensure agreement and fidelity of data collection and teacher implementation. The terminal goal for task completion was 90% or greater for each student. As the primary dependent measure, phase changes for all participants throughout the study were determined based on visual analysis of student task completion data. 

Off-task behavior was included as a secondary dependent variable to evaluate the relationship between increased task completion and reductions in off-task behavior, despite the fact that the contingencies for off-task behavior were never modified throughout the study. The researcher (first author) initially met with the teacher to define off-task behavior based on individualized student information. The teacher reported information about the topography of students’ off-task behavior and developed a behavioral definition with the researcher that encompassed all topographical variations of off-task behavior exhibited by each student. Off-task behavior was defined as engaging in tasks or attending to activities other than those instructed or assigned by the teacher. The off-task behavior was measured via direct observation using a 10-s-partial interval recording system, a pencil, and a mobile timer. Off-task behavior was scored if the student was (a) out of their assigned area of the classroom without permission from the teacher (out of the seat, standing or sitting more than 3 ft away from desk area), (b) speaking or otherwise interacting with others unrelated to task or instruction (talking, whispering, or gesturing to others without permission), (c) averts eyes from teacher, materials, instruction, or other task-related stimuli (staring into space, head on desk, looking at peers for 2 s or more), or (d) engaging in bodily movements interfering with assigned task (e.g., playing with pencil, ripping paper).

#### 2.3.2. Procedural Fidelity

Two types of procedural fidelity were assessed: fidelity of training procedures and teacher implementation fidelity. Following the baseline, the researcher conducted teacher training on implementing the technology-based SMP with reinforcement and on utilizing behavioral skills training procedures (BST; [35]), consisting of instruction, modeling, rehearsal, and feedback. The 31-item Fidelity Checklist for Teacher Training on Intervention Implementation and the 13-item Fidelity Checklist for Teacher Training on BST were used during teacher training to ensure accurate delivery of training. The fidelity of each teacher-implemented student training session was assessed by the researcher using the 8-item Fidelity Checklist for Student Training. Teacher implementation fidelity was also assessed for 100% of all intervention sessions using the Teacher Implementation Fidelity Checklist. The checklist included procedural components such as providing necessary materials, completing each step of the debriefing, and delivering the appropriate reinforcer after the session. Teacher implementation fidelity averaged 97% across participants in the intervention and fading phases ranging from 90–100% across students, which surpassed the goal of 90% on average. 

#### 2.3.3. Interobserver Agreement (IOA)

The researcher and one RA collected data. Both were graduate students enrolled in an Applied Behavior Analysis graduate program. Observer training was conducted prior to the study and consisted of researcher-implemented BST to teach the operational definitions and data collection procedures until the RA demonstrated proficiency in collecting data (IOA reached 95% or higher) on two consecutive rehearsals. The research team assessed IOA on student behaviors and procedural fidelity by simultaneously collecting data on student target behaviors and teacher and researcher fidelity using the same data sheets to assess IOA.

The IOA was assessed for 32.5% of observation sessions across participants and phases. For off-task behavior, IOA was calculated by dividing the total number of intervals with agreements by the total number of intervals with agreements plus disagreements and multiplying by 100. IOA for task completion was assessed by reviewing student work samples and dividing the total number of agreements (+) of SM checklist items validated as completed by the total number of SM checklist items with agreements plus disagreements for tasks marked as completed (+) and multiplying by 100. IOA never dropped below 90% during baseline, 94% during intervention, and 100% during fading. During baseline, the average IOA for off-task was 94% for Dominic, 97.5% for Selena, and 95.5% for Robin, ranging from 90–100%. IOA for task completion was 100% for all participants. During the intervention, the average IOA for off-task behavior was 99% for Dominic, 99.5% for Selena, and 97% for Robin, ranging from 94–100%. For task completion, IOA was 100% across all participants during the intervention. Finally, IOA was assessed for 33% of fading sessions for each participant. During fading, IOA was 100% across participants and behaviors. IOA for fidelity was assessed during 100% of intervention and fading sessions, which averaged 99% (range = 90–100%) for all participants across phases. 

#### 2.3.4. Social Validity 

Students and teacher participants and a school administrator completed a social validity questionnaire following the study’s completion. The teacher social validity questionnaire was adapted from the Intervention Rating Profile-15 (IRP-15; [36], which was rated on a 5-point Likert rating scale with 1 = strongly disagree and 5 = strongly agree. The students completed a researcher-developed 10-item questionnaire to evaluate their acceptability and enjoyment of the intervention and agreement that the SMP produced improvements to their academic performance and behavior. The teacher completed a 16-item Teacher Acceptability Questionnaire to evaluate the perception of goals of the intervention, acceptability of procedures, and likelihood to use SM interventions in the future. One school administrator completed a 4-item Administrator Acceptability Questionnaire assessing their perspective of the utility of technology-based SMP to support students in the general education setting.

### 2.4. Experimental Design and Procedures

This study used a concurrent multiple baseline design across participants to examine the effects of the technology-based SMP intervention. An ABC sequence was used to evaluate the SMP intervention consisting of three phases: baseline, SMP with differential reinforcement, and fading. The research team determined phase changes based on stable rates of task completion over four consecutive sessions. Stable responding in each phase was indicated by an absence of changes in the level of behavior or an observed countertherapeutic trend (during baseline only). 

#### 2.4.1. Baseline 

The baseline consisted of typical classroom instructional activities (e.g., ELA or Writing) with no SMP intervention in place. During baseline, teacher-implemented consequences and classroom management strategies remained in place with no alterations to the environment related to the study. The teacher responded as usual to off-task behavior based on the pre-existing procedures already established in the classroom environment (e.g., planned ignoring, time-out, brief verbal redirection). The research team collected data on the occurrence of off-task behavior and the percentage of task completion achieved throughout each baseline session.

#### 2.4.2. Teacher Training 

The researcher conducted two training sessions with the teacher following the baseline prior to intervention implementation. Each training was scheduled at the teacher’s convenience and lasted approximately 30 min. Teacher intervention training consisted of researcher-implemented BST to train the teacher to implement the technology-based SMP intervention according to the task-analyzed steps included within the implementation fidelity checklist. During role-play scenarios, the researcher acted as the student while the teacher rehearsed implementing the procedures using the study materials. The researcher provided feedback on the teacher’s performance and rehearsals continued until the teacher achieved the mastery criterion of 100% accuracy on two consecutive role-play scenarios. Following training, a pocket guide of the implementation checklist was given to the teacher to review and reference throughout the study. The pocket guide consisted of simplified (i.e., more concise, bullet points) versions of the detailed descriptions of each step included on the implementation fidelity checklist. The pocket guide was created as a resource for the teacher to refer to during implementation to increase the likelihood that each step of the checklist would be implemented as intended. When the teacher missed the same step on the fidelity checklist for two consecutive intervention sessions, booster training was conducted to review the missed step and provide opportunities to rehearse target skills. The booster training followed the same approach as the initial teacher training and involved implementing the BST procedures. The training consisted of researcher-implemented BST to train the teacher on how to use BST to train students on the SMP procedures. The BST training for teachers continued until the teacher demonstrated proficiency in implementing each component of BST with 100% accuracy on two consecutive role-play attempts. The researcher implemented both teacher intervention training and teacher BST training with 100% fidelity as measured by an RA.

#### 2.4.3. Student Training 

Student training sessions were implemented in a staggered fashion directly prior to the first intervention session for each student participant. These training sessions lasted for 30 min and involved the teacher using BST to teach the students how to participate in the SMP intervention. The teacher provided instructions for self-monitoring using the List app, modeled the procedures, role-played a typical session with the student, and provided feedback on their performance. During the role-plays, the teacher provided the materials to practice using the SMP procedures while completing abbreviated tasks for two consecutive 3 min practice sessions as the students practiced self-monitoring using the List app. Training continued until the students demonstrated each step of the SMP procedures with 100% accuracy in two consecutive role-plays. The researcher assessed the fidelity of student training conducted by the teacher for each student using a fidelity checklist. The assessment revealed that the training was implemented with 100% fidelity.

#### 2.4.4. SMP with Differential Reinforcement 

The technology-based SMP with reinforcement intervention was implemented in a staggered fashion concurrently across student participants. The SMP intervention was delivered to participants once a stable pattern of responding to student behaviors was demonstrated during baseline. The teacher continued to implement typical classroom management strategies during the intervention and no changes were made to the type, frequency, or intensity of the tasks presented during sessions. Implementation continued until each participant demonstrated stable changes in task completion with a separation in the data for five or more consecutive sessions. During SMP, students used the List app to monitor their task completion. Before each session, the teacher prepared and entered a list of tasks to be completed throughout the instructional period for each student. To begin the session, the teacher delivered the device and task materials and verified that the students understood the instructions for the tasks and criteria for reinforcement during the session. During sessions, students completed tasks as usual and checked the box next to each item on the List app as they completed the corresponding task. No prompts were delivered for students to self-assess and no modifications were made to the tasks presented or consequences for off-task during intervention. After the session, the teacher met with each student individually to debrief. The teacher validated student work samples by recording whether each task was completed, recorded the percentage of task completion on the debriefing form, confirmed the appropriate number of points earned, and provided feedback on the steps of SMP. The session concluded when the teacher delivered the differential reinforcement after debriefing.

Students received reinforcement during SMP based on the number of points earned throughout the academic period. Students could earn up to four points per session based on the percentage of task completion achieved and the accuracy of SMP. Students could earn one point for accurately recording their performance during the period. If the students did not accurately self-monitor for two consecutive sessions, the teacher conducted a brief booster training until mastery was demonstrated. Students could earn up to an additional three points based on the level of task completion achieved throughout each intervention session. The criterion levels were set by the teacher based on an average of student performance during baseline. For example, if a student demonstrated a baseline level of 50% task completion, they could earn three points for obtaining 75% task completion or higher. The points that students earned throughout the session were exchanged directly for backup reinforcers, which were always delivered at the end of the session. 

Backup reinforcers were categorized into four leveled tiers to correspond with the four possible points that a participant could earn per session. For example, if a student earned four points, they would choose from more highly preferred reinforcers (e.g., computer time, art activities, edibles) for the maximum amount of time (e.g., 10 min); whereas if a student earned two points, a lower preferred reinforcer (e.g., sticker, fidget toy) was available for a lesser amount of time (e.g., 2 min). During the debriefing, the teacher presented the student with available reinforcers from a reinforcer menu that contained all of the possible individualized reinforcers that a student could select from. Students selected an appropriate reinforcer for the number of points accumulated and the reinforcer was delivered following the session.

#### 2.4.5. Maintenance Evaluations 

A fading procedure consisting of increased delay to reinforcement was embedded into the intervention procedures to promote maintenance of increased task completion and reductions in off-task behavior. Students completed classroom tasks as usual using the technology-based SMP and debriefed with the teacher after each session. Differential reinforcement may be faded by systematically altering the quality, duration, immediacy, or magnitude of reinforcement may be a practical alternative in cases where extinction is not possible as suggested by Vollmer and colleagues (2020). Because each student reliably completed 100% of tasks presented during the initial intervention phase, fading consisted of a delay to the reinforcement of 45 min to up to 60 min (to the end of the instructional period). Fading was complete in the current study when increases in task completion persisted for at least three consecutive sessions for each student with stability demonstrated in the data.

### 2.5. Data Analysis

In addition to visual analyses, quantitative effect size estimates were computed using the percentage of goal obtained (PoGO [37]), which is a within-case parametric effect size index that compares observations to a determined goal [37,38]. It is calculated by dividing the distance from the mean or median value of the baseline to that of intervention by the distance from the mean or median value of the baseline to the goal line and multiplying by 100. When interpreting PoGO, Ferron et al. [37] suggest that effect size estimates ranging from 20 to 40 are considered a small effect, 40 to 60 a medium effect, 60 to 80 a moderately large effect, and a large effect when PoGO exceeds 80. 

## 3. Results

**Figure 1:**
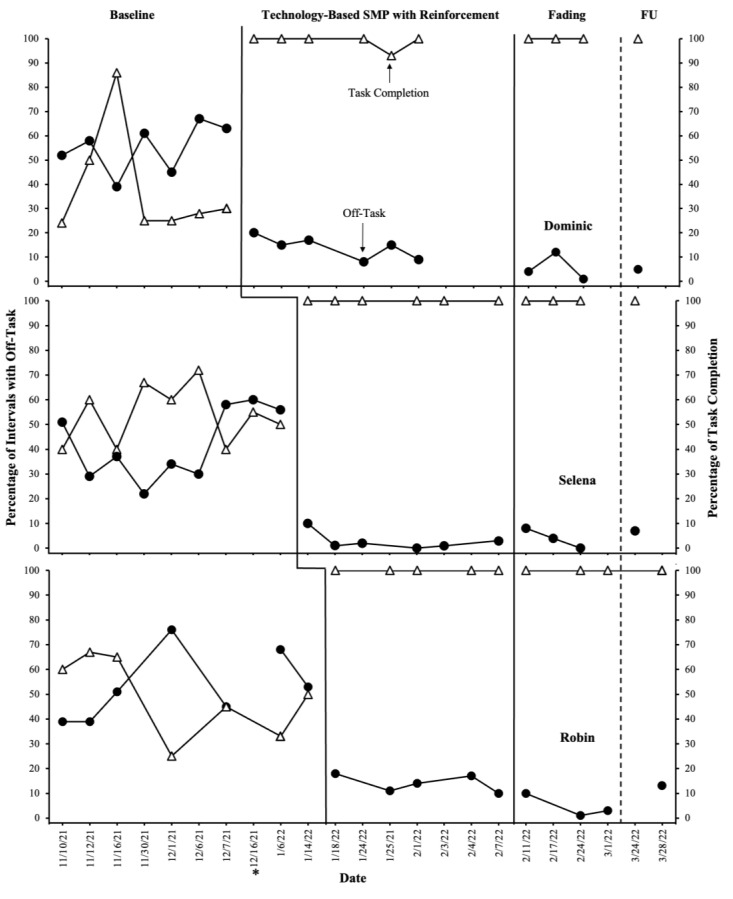
Percentage of intervals of off-task behavior and task completion across conditions and students. The asterisk on *x*-axis indicates a change in environment on a day when two classes were combined (double the number of students in the same classroom).

Figure 1 presents data on student behaviors across baseline, technology-based SMP with reinforcement, and fading for each participant. Overall, the results indicate that technology-based SMP was effective in increasing task completion above the terminal goal level while concurrently decreasing off-task behavior for each student. No overlap for either data path was observed between the baseline and intervention phases for Dominic, Selena, or Robin, indicating a positive demonstration of intervention effects. The improvements in student behavior maintained during fading in which a delay to reinforcement was implemented. Furthermore, reductions in off-task behavior and increases in task completion were maintained for each student during a 4-week follow-up. 

During baseline, Dominic exhibited a moderate to high level of off-task behavior (M = 55%; range = 39–67%) and a relatively stable low level of task completion (M = 38%; range = 24–86%). Implementation of the SMP intervention resulted in an immediate increase in task completion of up to 100% and an immediate decrease in off-task behavior, below that observed in any baseline session. Dominic consistently showed improvements across both behaviors throughout the intervention phase. Task completion demonstrated sustained increases, averaging at 99% (range = 93–100%), while off-task behavior exhibited slight decreases during sessions 8–12. Notably, off-task behavior remained consistently low and stable, with an average of 14% (range = 9–20%) throughout the intervention phase. Moreover, task completion rates reached 100% and off-task behavior continued to decrease (M = 5%; range = 1–12%) in each fading session and maintained at a 4-week follow-up. 

During baseline, Selena exhibited a moderate level of off-task behavior (M = 42%; range = 29–60%) and a slightly variable, moderate to low level of task completion (M = 54%; range = 40–72%). When the intervention was implemented for Dominic, a stable counter-theraputic level of task completion and off-task behavior persisted for Selena. However, upon implementation of the SMP intervention, Selena exhibited an immediate increase in task completion to 100% and a reduction in off-task behavior to 10%, below baseline levels, within the first intervention session.

Off-task behavior continued to decrease to near-zero levels throughout the intervention for Selena (range = 0–3%). During the intervention phase, Selena’s teacher anecdotally noted notable improvement in Selena’s academic progress, particularly in her reading comprehension level and descriptive writing abilities. When a delay in the reinforcement was introduced as part of the fading process, Selena consistently completed tasks without any decrease in performance, maintaining a 100% completion rate. Initially, there was a slight increase in off-task behavior, reaching 8%. However, it showed a decreasing trend in subsequent fading sessions, dropping to 4% and eventually reaching 0%. These positive changes in Selena’s behavior were maintained during the 4-week follow-up period, further demonstrating the effectiveness of the SMP intervention.

Finally, during baseline, Robin exhibited off-task behavior at a variable, high level (M = 53%; range = 39–76%) and a slightly variable, moderate to low level of task completion (M = 49%; range = 25–67%). These patterns persisted when intervention was introduced to Dominic and Selena, respectively. Similar treatment effects were observed for Robin upon implementation of the SMP intervention. Specifically, task completion increased to and remained at 100% during each intervention session while reductions in off-task behavior remained low and stable (M = 14%; range = 10–18%). Task completion remained at 100% during fading, while off-task behavior continued to decrease (M = 5%; range = 1–10%). Robin’s positive behavior changes, likewise, were maintained at a 4-week follow-up. In combination, these effects demonstrate a functional relationship between the SMP intervention and student behavior. 

During follow-up observations, the teacher continued to implement the intervention despite no longer being required to do so following the study. The continued implementation of the intervention reflects the high social validity of the intervention. Furthermore, during follow-up, the level of off-task remained at 5% for Dominic, 7% for Selena, and 13% for Robin while task completion remained at 100% for all students. These results demonstrate the utility of technology-based SMP in increasing task completion and reducing off-task behavior exhibited by students with disabilities in general education settings. Furthermore, the positive behavior changes maintained for each student during fading and follow-up, suggest that these procedures may yield positive behavior change that is both efficient and durable.

### 3.1. Effect Sizes

PoGO effect sizes for off-task behavior ranged from 74 to 93 (74, 93, and 74 for Dominic, Selena, and Robin, respectively) indicating a moderately large to large effect. For task completion, PoGO effect sizes ranged from 98 to 100, indicating a large effect. For Selena and Robin, PoGO effect sizes were 100, demonstrating that the goal was met.

### 3.2. Social Validity 

Social validity was evaluated after the final fading session by gathering feedback from student and teacher participants, as well as a school administrator who observed implementation on more than one occasion when the researcher was not present. Overall, students reported high levels of satisfaction with the intervention and procedures. On a 5-point Likert rating scale, ratings averaged 4.9 or 5.0 for each student. Near perfect ratings were given for each acceptability item, including the desire to use SMP in the future, enjoyment of procedures, and agreement that the SMP resulted in improvements to their academic performance and classroom behavior. Social validity assessments with the classroom teacher and school administrator were similarly promising, with near-perfect ratings for each treatment acceptability item. The average acceptability rating provided by the teacher and administrator was equally 4.8, with the lowest allocated score for any item being 4. The teacher reported high levels of satisfaction with the student outcomes and continued to use the intervention following the study. The teacher indicated that the procedures were easy to implement and produced immediate positive behavioral and academic effects for all students, piquing her interest in the possibility of utilizing the SMP as a class-wide intervention in the coming years. The administrator indicated high levels of satisfaction with student and teacher outcomes. The administrator also noted that she was pleased with the technology-based intervention application and likewise reported interest in various class-wide applications of SMP.

## 4. Discussion

This study evaluated the extent to which implementation of the technology-based SMP accompanied by differential reinforcement resulted in a reduction in off-task behavior and an increase in task completion among students with disabilities served in a general education classroom. This study also sought to examine where the effects of SMP intervention would maintain after the fading procedure was completed. The results of the study revealed that technology-based SMP with differential reinforcement produced marked reductions in off-task behavior and immediate increases in task completion, with all students achieving a 100% completion rate. In fact, the first intervention session for Dominic occurred during a non-typical classroom routine when two classes were combined. The tasks presented during the session remained the same, but approximately 20 additional students were present. Yet, an increase in task completion to 100% and a reduction in off-task behavior to 20% were demonstrated despite additional environmental distractions. 

The consistent decrease in off-task behavior observed in all three students is noteworthy, particularly because they were not required to routinely divert their attention from the classroom tasks that they were completing to record their attentional behaviors at pre-determined intervals. Despite this absence of consistent monitoring, reliable decreases in off-task behavior and unprecedented levels of task completion persisted for all students. Increases in task completion were equally notable; during the intervention, each student completed 100% of the tasks presented to them in 96% of intervention and fading sessions. The classroom teacher anecdotally noted during the study that participating students consistently outperformed their same-age peers regardless of disability in terms of task completion achieved while using SMP. Specifically, students were only required to complete up to 90% of their work at any point in time to earn the maximum three points for task completion, yet students reliably obtained more than the minimum performance criteria by achieving 95% or more task completion during each SMP session. Moreover, compared to the variability of task completion in baseline, the repeated demonstration of task completion at 100% during intervention illustrates a clear functional relation. Increases in task completion and reductions in off-task behavior were maintained during fading, even when the criteria for reinforcement increased and a delay to the reinforcement of 45 min or more was implemented. 

These results are in alignment with previous research suggesting that teaching students to self-monitor academic behavior may result in better academic and social outcomes as compared to teaching students to self-monitor attentional behavior [13]. These results suggest that technology-based SMP is contextually fit for school settings because the teacher did not have to collect data, modify existing classroom management strategies, or alter established classroom routines to implement. The intervention was socially validated by students and teachers alike and had immediate positive effects on student behavior and performance. These findings are consistent with previous research that highlights the effectiveness of SMP in improving the academic performance of students with disabilities [13] and that emphasizes the role of reinforcement in enhancing the effectiveness of SM intervention [30]. Lastly, this study extends the literature suggesting that technology-based intervention applications may be highly motivating to students while still producing positive effects on behavior and academic performance [24,32,33].

Several overarching implications for practice should be noted. First, the SMP intervention may be a viable classroom intervention to promote increased task completion and reductions in off-task behavior for a diverse range of students with complex academic and behavioral needs. The student participants in the current study exhibited a range of externalizing and internalizing problem behaviors that hindered their engagement in classroom instruction. It is notable that the same intervention procedures produced substantial positive changes for each student despite differences in their academic and behavioral needs. This suggests that teachers may utilize a general SMP procedure in the classroom setting to support a wide range of students with diverse behavioral needs. Additionally, teachers may implement the SMP intervention with multiple students displaying problem behavior and/or inadequate levels of task completion simultaneously in the classroom setting with relative ease. Therefore, the SMP intervention may be a feasible class-wide or targeted intervention to address off-task behavior exhibited in the classroom with little investment of teacher time or resources. 

While the current study shows promising results, some limitations have been identified. The first limitation relates to the small sample size of students included in the study. With time constraints and difficulties navigating the COVID-19 pandemic, a small number of participants opted to participate in the study. Other common limitations in academic settings occurred during this study (e.g., student suspensions, state testing, and changes in classroom routines), which restricted data collection opportunities and could have potentially impacted the data set. Restricted opportunities for data collection paired with a limited sample size may limit the generalizability of the current findings. Future researchers could replicate this study with a larger, more diverse sample to evaluate the external validity of the current findings. Furthermore, evaluating the intervention effects when used with different populations of students with various complex behavioral needs would promote a more robust dissemination of effective EBPs for general education teachers. 

In alignment with the first limitation, the inclusion of only one general education teacher within the study may limit the generalizability of the results. The intervention was implemented by the same teacher for all participating students during the same instructional routines. The teacher used the same classroom management strategies, and instructional techniques, and evaluated the students using identical tasks and task lists. Future research should evaluate the technology-based SMP with differential reinforcement intervention when implemented by different teachers in various settings during alternate routines to improve the external validity of the findings. Identifying alternative reinforcement procedures and adaptations to the SMP intervention may promote the generalized utility within complex settings, such as the inclusive classroom. 

Finally, teacher implementation fidelity during the first three intervention sessions was 91% across students (range = 80–100%) despite the teacher having demonstrated 100% accuracy in implementing steps on multiple consecutive attempts during training. After missing the same step on the fidelity checklist for two consecutive sessions, the teacher received booster training. Following booster training, the teacher missed the step again during Session 2 when the intervention was implemented for Dominic and Selena; therefore, the researcher conducted two 5 min in vivo coaching sessions. After coaching, implementation fidelity increased to and remained at 100%. These findings suggest that in vivo coaching may be necessary to support teachers to implement classroom interventions with high fidelity following training. Even without lapses in fidelity, in vivo coaching may enhance the effectiveness of the training and promote improved fidelity beyond training circumstances. 

The fading procedure implemented in this study demonstrated promising results as improvements in student target behaviors were maintained with a delay in reinforcement. However, it is worth noting that some components (e.g., SM checklist, points system, reinforcement) of the intervention remained in place during this condition. Although the continued implementation of some intervention components reflects the high social validity of the intervention, it is worth considering that they may have inadvertently influenced the data collected during fading. Furthermore, while access to backup reinforcers was delayed during fading, teacher attention was still provided without delay, which may have functioned as a reinforcer for some participants during the fading phase. Future researchers should evaluate the maintenance effects after completely removing the intervention to improve the strength of the evidence. 

Additionally, with 1:1 technology policies in public schools on the rise, technology-based applications of interventions are increasingly relevant and may have valuable applications for a large number of students in school-based settings. Our study suggests that additional experimental research evaluating the effects of technology-based SM to improve student behavior is warranted [15,32]. Future researchers may also evaluate the technology-based SMP with reinforcement when implemented as a class-wide intervention in a general education setting as expressed by the teacher who participated in the study. Furthermore, future researchers should evaluate the differential effectiveness of specific recording mediums for SM, such as high-tech, low-tech, and no- tech (pencil and paper) methods, to determine which strategies may be more socially valid and/or preferable for use by teachers. Moreover, a comparison of recording methods may allow for recommendations of which procedures produce the greatest improvements in student behavior. Finally, it remains unclear if the current result may have differed if the additional instruction during the teacher debriefing had not been included as a component of the SMP intervention. It is possible that the high fidelity of the other components (i.e., reinforcement, teacher, and student rating accuracy) could have been adequate to obtain the same results. Consistent with previous research [26], the current investigation suggests that future researchers should examine the moderating effects of various treatment components (i.e., reinforcement, feedback, debriefing) to determine the maximally effective treatment arrangements for students with disabilities. Future researchers may also consider evaluating the intervention with other student populations in other settings. 

In conclusion, this study offers evidence that the technology-based SMP with differential reinforcement can be a feasible, practical, and effective intervention to increase task completion and reduce off-task behavior in students with disabilities served in a general education classroom. The efficiency of the intervention and immediacy of the results suggest that the technology-based SMP with differential reinforcement may be a proactive classroom intervention for use by general education teachers to support students with disabilities in their classrooms. The technology-based SMP augmented with reinforcement may also promote the development of self-regulation skills in students with disabilities, which are crucial for improving their long-term social, behavioral, and academic outcomes.

## Data Availability

Data are available on reasonable request from the corresponding author.
